# Spectrophotometric Determination of Mycophenolate Mofetil as Its Charge-Transfer Complexes with Two *π*-Acceptors

**DOI:** 10.1155/2012/875942

**Published:** 2012-03-26

**Authors:** K. B. Vinay, H. D. Revanasiddappa, M. S. Raghu, Sameer. A. M. Abdulrahman, N. Rajendraprasad

**Affiliations:** Department of Chemistry, University of Mysore, Manasagangotri, Mysore 570 006, India

## Abstract

Two simple, selective, and rapid spectrophotometric methods are described for the determination of mycophenolate mofetil (MPM) in pure form and in tablets. Both methods are based on charge-transfer complexation reaction of MPM with p-chloranilic acid (p-CA) or 2,3-dichloro-5,6-dicyano-1,4-benzoquinone (DDQ) in dioxane-acetonitrile medium resulting in coloured product measurable at 520 nm (p-CA) or 580 nm (DDQ). Beer's law is obeyed over the concentration ranges of 40–400 and 12–120 *μ*g mL^−1^ MPM for p-CA and DDQ, respectively, with correlation coefficients (*r*) of 0.9995 and 0.9947. The apparent molar absorptivity values are calculated to be 1.06 × 10^3^ and 3.87 × 10^3^ L mol^−1^ cm^−1^, respectively, and the corresponding Sandell's sensitivities are 0.4106 and 0.1119 *μ*g cm^−1^. The limits of detection (LOD) and quantification (LOQ) are also reported for both methods. The described methods were successfully applied to the determination of MPM in tablets. Statistical comparison of the results with those of the reference method showed excellent agreement. No interference was observed from the common excipients present in tablets. Both methods were validated statistically for accuracy and precision. The accuracy and reliability of the methods were further ascertained by recovery studies *via* standard addition procedure.

## 1. Introduction

Mycophenolate mofetil (MPM), chemically known as 2-morpholinoethyl (E)-6-(1,3-dihydro-4-hydroxy-6-methoxy-7-methyl-3-oxo-5-isobenzofuranyl)-4-methyl-4-hexenoate, is an immunosuppressive agent, inosine monophosphate dehydrogenase (IMPDH) inhibitor. MPM is rapidly absorbed following oral administration and hydrolysed to its metabolite, mycophenolic acid (MPA). MPA as a newer immunosuppressant is mainly used in acute rejection treatment [1].

A survey of literature revealed that a few chromatographic methods have been reported to quantify mycophenolic acid (MPA) and its degradation products in body fluids [2–6]. Sugioka et al. [7] have developed high-performance liquid chromatographic (HPLC) method for the determination of MPM in biological samples including rat and human body fluids. However, there are only three reports on the determination of MPM in pharmaceuticals. Protic et al. [8] have applied HPLC for the simultaneous determination of MPM and its degradation product MPA in dosage form. An HPLC method for detecting dissolution of MPM capsules and assay of the drug has been developed by Yang and He [9]. The method is reported to be applicable over a concentration range 62−621 *μ*g mL^−1^ MPM. Recently, two UV-spectrophotometric methods to determine MPM in 5−40 *μ*g mL^−1^ range have been reported by Verma et al. [10]. The absorbance of MPM in either 0.1 M HCl or acetate buffer of pH 4.9 was measured at 250 nm.

Molecular interactions between electron donors and acceptors are generally associated with the formation of intensely colored charge-transfer (CT) complexes which absorb radiation in the visible region [11, 12]. Substituted quinones such as 2,3-dichloro-5,6-dicyano-1,4-benzoquinone (DDQ) and p-chloranilic acid (p-CA) have been used as *π*-acceptors with various donors to form C-T complexes and radicals. Applications of p-CA and DDQ for the determination of a number of pharmaceutical compounds [13–18] have recently been described.

To the best of our knowledge, no visible spectrophotometric method is available for the quantification of MPM in pharmaceuticals. This paper describes two visible spectrophotometric methods based on the interaction between the amine moiety of MPM as the n-donor and p-CA as well as DDQ as *π*-acceptors resulting in the formation of intensely coloured C-T complexes. The selectivity, sensitivity, accuracy, and precision of the developed methods, and their application to the determination of MPM in tablets were studied.

## 2. Experimental

### 2.1. Apparatus

All absorption measurements were made using a Systronics model 106 digital spectrophotometer (Systronics Ltd., Ahmadabad, India) with 1 cm path length quartz cells.

### 2.2. Materials

Pharmaceutical grade MPM was procured from Apotex Pvt. Ltd., Bangalore, India, and certified to be 99.5% pure. It was used without further purification. CellCept 500 (Roche, Mumbai, India) tablets were purchased from local market. 1,4-Dioxane and acetonitrile (spectroscopic grade) were purchased from Merck, Mumbai, India. Distilled water was used wherever required. All other chemicals used were of analytical reagent grade.

### 2.3. Reagents

A 0.5% p-chloranilic acid (p-CA) and 0.25% 2,3-dichloro-5,6-dicyano benzoquinone (DDQ) (both from S. D. Fine Chem. Ltd., Mumbai), respectively, were prepared freshly in 1,4-dioxane.

 For p-CA method, a 500 *μ*g mL^−1^ MPM stock solution was prepared by dissolving 50 mg of pure drug in acetonitrile in a 100 mL volumetric flask, the solution was diluted to the mark with the same solvent, and the above 500 *μ*g mL^−1^ MPM stock solution was diluted with acetonitrile to get 150 *μ*g mL^−1^ and used for the assay in DDQ method.

### 2.4. Assay Procedure

#### 2.4.1. p-CA Method

Varying aliquots of standard MPM solution (0.4–4.0 mL of 500 *μ*g mL^−1^) equivalent to 40–400 *μ*g mL^−1^ were accurately transferred into a series of 5 mL calibrated flasks and the total volume in each flask was brought to 4 mL by adding acetonitrile. After the addition of 1 mL of 0.5% p-CA solution, the content was mixed well and the absorbance was measured after 5 min at 520 nm against a reagent blank similarly prepared without adding MPM solution.

#### 2.4.2. DDQ Method

Into a series of 5 mL calibration flasks, aliquots (0.2–4.0 mL) of standard MPM solution (150 *μ*g mL^−1^) equivalent to 6–120 *μ*g mL^−1^ MPM were accurately transferred, and to each flask 1 mL of 0.25% DDQ solution was added and mixed. After 5 min, the absorbance of the purple coloured was measured at 580 nm against the reference blank similarly prepared without drug. 

Standard graph was prepared by plotting the absorbance *versus* MPM concentration, and the concentration of the unknown was read from the calibration graph or computed from the respective regression equation derived using the absorbance-concentration data.

#### 2.4.3. Procedure for Commercial Dosage Forms

Twenty tablets were weighed and pulverized. An amount of tablet powder equivalent to 50 mg MPM was transferred into a 100 mL volumetric flask and about 70 mL of acetonitrile was added to the flask. The content was shaken well for 20 min and diluted to the mark with the same solvent. The resulting solution was filtered through Whatmann number 42 filter paper and used for the assay by following the general procedure described for p-CA method. This tablet extract (500 *μ*g mL^−1^) was diluted to 150 *μ*g mL^−1^ with acetonitrile and a suitable aliquot was used for the assay in DDQ method.

#### 2.4.4. Procedure for the Analysis of Placebo Blank and Synthetic Mixture

A placebo blank containing starch (15 mg), acacia (10 mg), sodium citrate (10 mg), hydroxyl cellulose (10 mg), magnesium stearate (10 mg), talc (15 mg), and sodium alginate (10 mg) was prepared by mixing all the components into a homogeneous mixture. A 5 mg of the placebo blank was accurately weighed and its solution was prepared as described under “Procedure for commercial dosage forms” and then subjected to analysis following the general procedures.

An accurately weighed 100 mg of MPM was added to 200 mg of placebo blank and homogenized. An amount of synthetic mixture containing 50 mg MPM was accurately weighed and transferred into a 100 mL volumetric flask and the extract equivalent to 500 *μ*g mL^−1^ MPM was prepared as described under “Procedure for commercial dosage forms” and used in p-CA method. Required volume of the previous extract was diluted to 150 *μ*g mL^−1^ in MPM with acetonitrile and used for DDQ method following the general recommended procedure.

## 3. Results and Discussion

### 3.1. Spectral Characteristics and Reaction Mechanism

The charge transfer complex forming reactions occur when *π*-acceptors react with the basic nitrogenous compounds which act as n-donors. Charge-transfer complex formation is characterized by electronic transition(s) to an excited state in which there is a partial transfer of electronic charge from the donor to the acceptor moiety. As a result, the excitation energy of this resonance occurs very frequently in the visible region of the electromagnetic spectrum. This produces usually intense-coloured characteristics for these complexes. Therefore, MPM, a nitrogenous base acting as n-donor, was made to react with p-CA and DDQ (*π*-acceptors) to produce a coloured charge transfer complexes in 1,4-dioxane-acetonitrile solvent system according to the following equation:

(1) $$\text{MPM}+\text{A}⇌\underset{\text{C-T complex}}{\text{MPM-A}}⇌{{\text{MPM}}^{∙}}^{+}+\underset{\text{Radical}\;\text{anion}}{{{\text{A}}^{∙}}^{-}}$$

**Figure 1:**
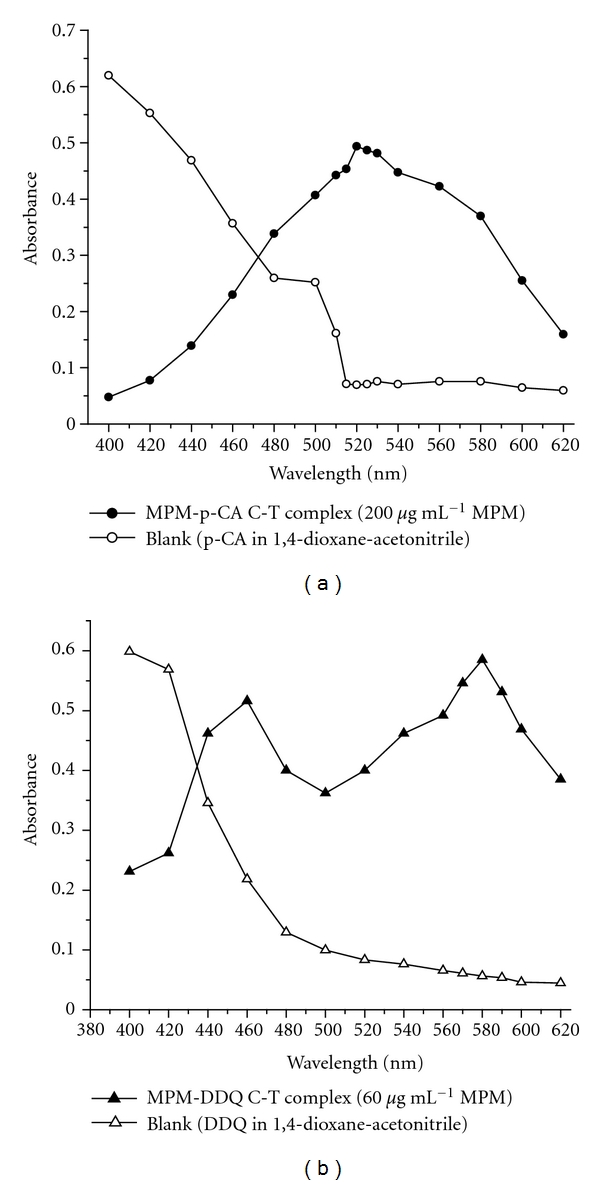
Absorption spectra of (a) MPM-p-CA C-T complex and its blank and (b) MPM-DDQ C-T complex and its blank.

In the p-CA method, MPM reacts with the reagent and gives a red chromogen that exhibits a strong absorption maximum at 520 nm in dioxane-acetonitrile medium (Figure 1(a)). This can be attributed to the formation of charge-transfer complex between MPM and p-CA followed by the formation of radical ions which probably was due to the dissociation of the original (MPM-p-CA) complex promoted by the high ionizing power of the acetonitrile solvent [19].

In the second method, the interaction of MPM with DDQ in dioxane-acetonitrile at room temperature gave a red-colored chromogen with strong absorption maxima at 460, 540, and 580 nm due to the formation of the free radical anion [20] and the wavelength 580 was selected for further studies because of higher sample absorbance and lower blank absorbance readings (Figure 1(b)).

### 3.2. Optimization of Reaction Conditions

Optimum conditions were established by measuring the absorbance of C-T complexes at 520 and 580 nm, for p-CA and DDQ method, respectively, by varying one and fixing other parameters.

#### 3.2.1. Effect of Reagent Concentration

**Figure 2:**
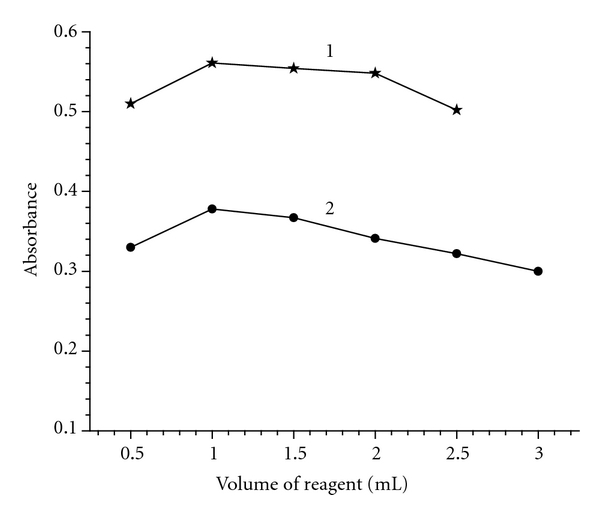
Effect of reagents concentration on color development: (1) p-CA (0.5%) using 200 *μ*g mL^−1^ MPM, and (2) DDQ (0.25%) using 40 *μ*g mL^−1^ MPM.

To establish optimum concentrations of the reagents for the sensitive and rapid formation of the MPM charge transfer complexes, the drug (MPM) was allowed to react with different volumes of the reagents (0.5–2.5 mL of 0.5% p-CA and 0.5–3 mL of 0.25% DDQ). In both the cases, maximum and minimum absorbance values were obtained for sample and blank, respectively, only when 1 mL of the reagent was used. Therefore, 1 mL of each reagent in a total volume of 5 mL was used throughout the investigation (Figure 2).

#### 3.2.2. Effect of Solvent to Dissolve Drug and Reagents

To dissolve MPM, acetonitrile was preferred to chloroform, dichloromethane, acetone, 2-propanol, 1,2-dichloroethane, 1,4-dioxane, methanol, and ethanol because as the complex formed in these solvents either had very low absorbance values or precipitated upon dilution, whereas in the case, of reagents, highly intense-coloured products were formed when 1,4-dioxane medium was maintained as solvent to dissolve p-CA and DDQ. Therefore, acetonitrile and 1,4-dioxane were chosen as solvents to dissolve MPM and the reagents, respectively.

#### 3.2.3. Effect of Reaction Time and Stability of the C-T Complexes

**Figure 3:**
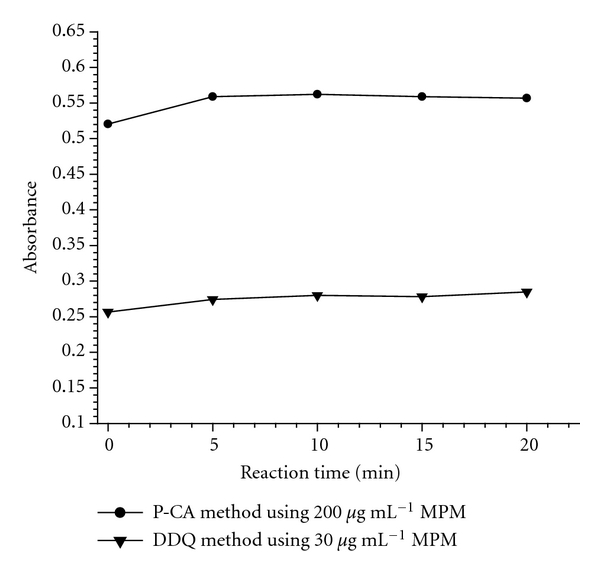
Effect of reaction time.

The optimum reaction times were determined by measuring the absorbance of the formed complex upon the addition of reagent solution to MPM solution at room temperature (Figure 3). In both methods, the formation of C-T complex was completed within 5 min and the absorbance values of the measured species were stable for 5 h and 20 min for p-CA and DDQ methods, respectively.

### 3.3. Investigation of Composition of C-T Complexes

**Figure 4:**
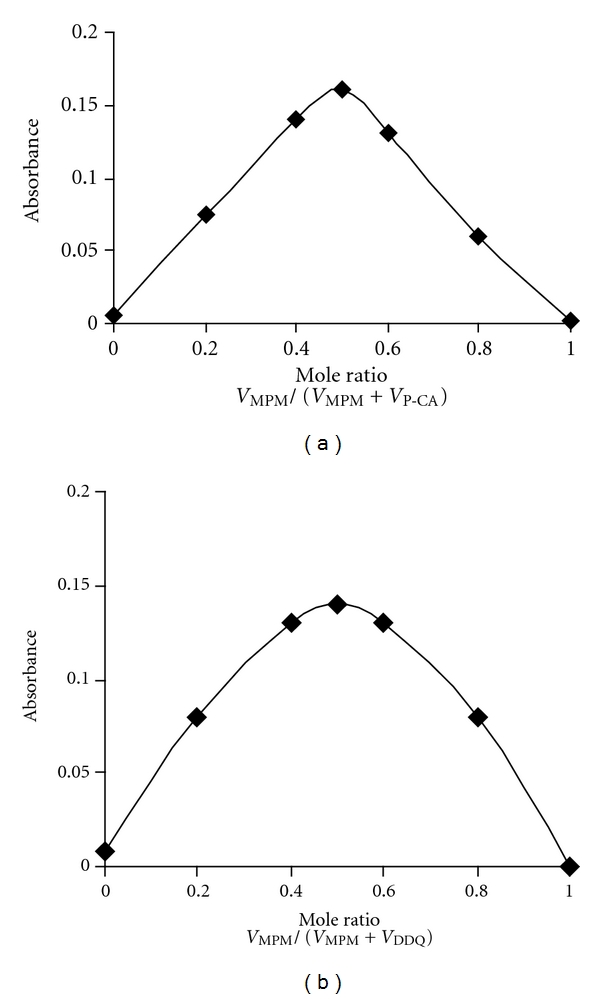
Job's plots obtained for (a) 4.61 × 10^-4 ^M MPM and p-CA C-T complex and (b) 2.31 × 10^−4^ M MPM and DDQ C-T complex.

**Scheme 1:**
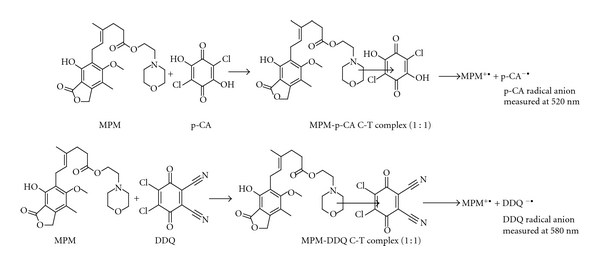
Proposed reaction path way for the formation of C-T complex between MPM and p-CA/DDQ.

The composition of the C-T complexes with either p-CA or DDQ was evaluated by following Job's continuous variations method [21]. The experiments were performed by preparing and mixing equimolar solutions of drug and reagent (p-CA method: 4.61 × 10^-4 ^M MPM and p-CA; DDQ method: 2.31 × 10^-4 ^M) by maintaining the total volume at 2.5 mL. The plots of the molar ratio between drug and reagent *versus* the absorbance values were prepared (Figures 4(a) and 4(b)), and the results revealed that the formation of C-T complex between drug and reagent followed a 1 : 1 reaction stoichiometry. This finding was anticipated by the presence of one basic electron donating center (nitrogen atom) in the MPM structure. Based on this fact, the following reaction pathway for the formation of C-T complex is proposed and shown in Scheme 1. The conditional stability constants (*K*_*f*_) of the charge transfer complexes were calculated [22] from the data of continuous variations method and found to be 9.46 × 10^6^ and 7.48 × 10^5^ for MPM-p-CA and MPM-DDQ complexes, respectively.

### 3.4. Association Constants and Free Energy Change

The absorbances of MPM-p-CA and MPM-DDQ complexes can be used to calculate the association constant using the Benesi-Hildebrand equation which depends on the experimental condition that one of the two component species should be present in large excess, so that its concentration is virtually unaltered on formation of the C-T complex.

In this study, the donor-acceptor systems were studied within a limited range of concentration. The acceptor concentrations were not low enough to be considered negligible with respect to donor concentration; the Ross and Labes equation was therefore used to calculate the association constant of the charge transfer complexes [23]:

(2) $$\frac{\left[A\right]\left[D\right]}{\left[A\right]+\left[D\right]}\times \frac{1}{A_{\lambda }}=\frac{1}{K\varepsilon_{\lambda }}\times \frac{1}{\left[A\right]+\left[D\right]}+\frac{1}{\varepsilon_{\lambda }},$$

where [*A*] and [*D*] refer to the molar concentration of acceptor and donor, respectively, *A*_*λ*_ is the absorbance at the wavelength *λ*, and *K* and *ε*_*λ*_ are the association constant and molar absorptivity, respectively. The association constants (*K*) for MPM-p-CA and MPM-DDQ complexes were calculated and found to be 1.096 × 10^4^ and 1.117 × 10^4^, respectively.

The standard free energy change of the complexation (Δ*G* ^0^) was calculated from the association constant (*K*) values according to the following well-established equation:

(3) $$\Delta G^{0}=-2.303RT\log K,$$

where Δ*G* ^0^ is the standard free energy of the C-T complex (kJ mol^−1^), *R* is the gas constant (8.314 J mol^-1 ^K^−1^), and *T* is the absolute temperature (298.15 K). The standard free energy change was calculated to be −23.06 and −23.11 kJ mol^−1^ for MPM-p-CA and MPM-DDQ complexes, respectively.

### 3.5. Method Validation

#### 3.5.1. Linearity, Sensitivity, Limits of Detection, and Quantification

A linear correlation was found between absorbance at *λ*_max⁡_ and concentration of MPM in the ranges given in Table 1. Regression analysis of Beer's law data using the method of least squares was made to evaluate the slope (b), intercept (a), standard deviations of *y*-axis, slope and intercept, and correlation coefficient (*r*) for each system and the values are presented in Table 1. The optical characteristics such as Beer's law limits, molar absorptivity, and Sandell sensitivity values [24] of both methods are also given in Table 1. The limits of detection (LOD) and quantitation (LOQ) calculated according to ICH guidelines [25] are also presented in Table 1. The moderate values of *ε* and Sandell sensitivity and LOD indicate the sensitivity of the proposed methods.

#### 3.5.2. Precision and Accuracy

The assays described under the construction of calibrations curves were repeated seven times within the day to determine the intraday precision and five times on different days to determine the interday precision of the methods. These assays were performed for three levels of analyte. The results of this study are summarized in Table 2. The percentage relative standard deviation (%RSD) values were ≤0.99% (intraday) and ≤1.78% (interday) indicating high precision of the methods. Accuracy was evaluated as percentage relative error (%RE) between the measured mean concentrations and taken concentrations for MPM. The percentage relative error was calculated at each concentration and these results are also presented in Table 2. Percent relative error (%RE) values of ≤3.04% demonstrates the high accuracy of the proposed methods.

#### 3.5.3. Selectivity

The results obtained from placebo blank and synthetic mixture analyses revealed that the inactive ingredients used in the preparation did not interfere in the assay of active ingredient. The absorbance values obtained from the placebo blank solution were almost equal to the absorbance of the blank which revealed no interference from the adjuvants. To study the role of additives added to the synthetic sample, 3 mL of the resulting solution prepared using synthetic mixture (500 and 150 *μ*g mL^−1^ in MPM from p-CA method and DDQ method) was assayed (*n* = 4). The percentage recoveries of 97.63–106.1 with %RSD values in the range 1.22–1.85 demonstrated the accuracy as well as the precision of the proposed methods and complement the findings of the placebo blank analysis with respect to selectivity. 

#### 3.5.4. Robustness and Ruggedness

The robustness of the methods was evaluated by making small incremental changes in the volume of reagent and contact time, and the effect of the changes was studied on the absorbance of the measured species. The changes had negligible influence on the results as revealed by small intermediate precision values expressed as %RSD (≤1.36%). Method ruggedness was demonstrated having the analysis done by four analysts and also by a single analyst performing analysis on four different instruments in the same laboratory. Intermediate precision values (%RSD) in both instances were in the range 0.54–3.15% indicating acceptable ruggedness. The results are presented in Table 3. 

#### 3.5.5. Application

The proposed methods were applied to the quantification of MPM in commercially available CellCept 500 tablets. The results obtained were compared with those obtained using a reference non aqueous potentiometric method [26] which is an official potentiometric titration of MPM with 0.1 M perchloric acid in anhydrous acetic acid medium. Statistical analysis of the results did not detect any significant difference in the performance of the proposed method to the reference method with respect to accuracy and precision as revealed by the Student's *t*-value and variance ratio *F*-value [27]. The results of this study are given in Table 4.

#### 3.5.6. Recovery Study

To further assess the accuracy of the proposed methods, recovery experiment was performed by applying the standard-addition technique. The recovery was assessed by determining the agreement between the measured standard concentration and added known concentration to the sample. The test was done by spiking the pre-analyzed tablet powder with pure MPM at three different levels (50, 100, and 150%, of the content present in the tablet powder (taken) and the total was found by the proposed method. Each test was repeated three times. From this test, the percentage recovery values were found in the range of 97.37–104.1 with standard deviation values from 0.74 to 1.08%. Closeness of the results to 100% showed the fairly good accuracy of the proposed methods. These results are shown in Table 5.

## 4. Conclusions

 Two simple, moderately sensitive, extraction-free, rapid, and cost-effective spectrophotometric methods based on charge transfer complex formation reaction were developed and validated for the determination of MPM. The reagents utilized in the proposed methods are cheap, readily available, and the procedure does not involve any critical reaction conditions or tedious sample preparation. The proposed methods are characterized by the wide linear dynamic ranges relative to narrow ranges (10–30 *μ*g mL^−1^) of the reported UV-spectrophotometric methods [10]. Also, the proposed methods are subject to no interference since the measurements are made at 520 and 580 nm compared to the reported methods [10] where the absorbance is measured at 250 nm and the chances of interferences from inactive ingredients are more there by affecting the accuracy of the results. The statistical parameters and the recovery data reveal good accuracy and precision of the methods. These methods can be used as general methods for the determination of MPM in bulk powder and tablets. The methods have many advantages over the separation techniques such as HPLC and include reduced cost and speed with high accuracy. Hence, the methods can be used in routine analysis of drug in quality control laboratories.

## Figures and Tables

**Table 1:** Sensitivity and regression parameters of the proposed methods in comparison with the reported UV-spectrophotometric methods [10].

Parameter	p-CA method	DDQ method	0.1 N HCl method	Acetate buffer method
*λ*_max⁡_, nm	520	580	250	250
Color stability	5 h	20 min	—	—
Linear range, *μ*g mL^−1^	40–400	6–120	10–30	10–30
Molar absorptivity (*ε*), L mol^-1 ^cm^−1^	1.06 × 10^3^	3.87 × 10^3^	NR***	NR
Sandell sensitivity*, *μ*g cm^−2^	0.4106	0.1119	NR	NR
Limit of detection (LOD), *μ*g mL^−1^	3.96	0.79	NR	NR
Limit of quantification (LOQ), *μ*g mL^−1^	11.99	2.40	NR	NR
Regression equation, Y**				
Intercept (a)	0.0100	0.0376	0.00316	0.01547
Slope (b)	0.0024	0.0080	0.02201	0.02168
Standard deviation of *y*-axis (*S*_*y*_)	0.0118	0.0416	—	—
Standard deviation of a (*S*_a_)	0.0145	0.0350	—	—
Standard deviation of b (*S*_b_)	5.3 × 10^−5^	4.75 × 10^−4^	—	—
Correlation coefficient (*r*)	0.9995	0.9947	0.9999	0.9996

*Limit of determination as the weight in *μ*g per mL of solution, which corresponds to an absorbance of *A* = 0.001 measured in a cuvette of cross-sectional area 1 cm^2^ and l = 1 cm. **Y = a + b*X*, where *Y* is the absorbance, *X* is concentration in *μ*g/mL, a is intercept, and b is slope. ***NR: not reported.

**Table 2:** Evaluation of intraday and interday accuracy and precision.

Method	MPM taken, *μ*g mL^−1^	Intraday accuracy and precision (*n* = 7)			Interday accuracy and precision (*n* = 5)		
		MPM found, *μ*g mL^−1^	%RE	%RSD	MPM found, *μ*g mL^−1^	%RE	%RSD
A	100.0	100.63	0.63	0.66	101.26	1.26	1.14
	200.0	200.22	0.11	0.36	201.28	0.64	0.78
	300.0	306.96	2.32	0.99	305.34	1.78	1.28

B	45.00	43.84	2.58	0.50	45.75	1.67	0.74
	75.00	76.04	1.39	0.87	76.69	2.25	1.36
	105.0	108.02	2.88	0.92	101.81	3.04	1.78

%RE: percent relative error; %RSD: relative standard deviation.

**Table 3:** Method robustness and ruggedness expressed as intermediate precision (%RSD).

Method	MPM taken, *μ*g mL^−1^	Robustness		Ruggedness	
		Parameters altered		Interanalysts (%RSD), (*n* = 4)	Interinstruments (%RSD), (*n* = 4)
		Volume of p-CA/DDQ*	Reaction time^Ψ^		
A	100.0	0.94	0.58	1.28	2.42
	200.0	1.36	0.65	0.84	3.15
	300.0	1.27	0.42	0.85	2.76

B	45.0	0.66	0.36	0.96	1.98
	75.0	0.74	0.85	0.78	2.38
	105.0	1.03	0.64	0.54	1.62

*The volumes of p-CA or DDQ added were 1 ± 0.2.

^Ψ^The reaction times were 5 ± 1 min.

**Table 4:** Results of analysis of CellCept 500 tablets^Ψ^ by the proposed methods and statistical comparison of the results with the reference method.

Nominal amount (mg/tablet)	Found* (percent of label claim ± SD)		
	Reference method	p-CA method	DDQ method
		101.0 ± 1.16	99.89 ± 1.74
500	100.6 ± 0.76	*t* = 0.66	*t* = 0.89
		*F* = 2.33	*F* = 5.24

*Mean value of 5 determinations.

Tabulated *t*-value at the 95% confidence level and for four degrees of freedom is 2.77.

Tabulated *F*-value at the 95% confidence level and for four degrees of freedom is 6.39.

^Ψ^Marketed by Sun pharmaceuticals.

**Table 5:** Results of recovery study *via *standard-addition method with CellCept 500.

p-CA method				DDQ method			
MPM in tablet, *μ*g mL^−1^	Pure MPM added, *μ*g mL^−1^	Total found, *μ*g mL^−1^	Pure MPM recovered (Percent ± SD*)	MPM in tablet, *μ*g mL^−1^	Pure MPM added, *μ*g mL^−1^	Total found, *μ*g mL^−1^	Pure MPM recovered (Percent ± SD)
101.0	50.0	152.8	103.6 ± 0.74	40.0	20.0	59.47	97.37 ± 0.76
101.0	100.0	202.5	101.5 ± 0.86	40.0	40.0	80.60	101.5 ± 1.08
101.0	150.0	249.1	98.74 ± 0.92	40.0	60.0	102.46	104.1 ± 0.84

*Mean value of three determinations.
